# The Prorenin and (Pro)renin Receptor: New Players in the Brain Renin-Angiotensin System?

**DOI:** 10.1155/2012/290635

**Published:** 2012-12-18

**Authors:** Wencheng Li, Hua Peng, Dale M. Seth, Yumei Feng

**Affiliations:** Department of Physiology, Tulane Hypertension and Renal Center of Excellence, Tulane University School of Medicine, 1430 Tulane Avenue SL-39, New Orleans, LA 70112, USA

## Abstract

It is well known that the brain renin-angiotensin (RAS) system plays an essential role in the development of hypertension, mainly through the modulation of autonomic activities and vasopressin release. However, how the brain synthesizes angiotensin (Ang) II has been a debate for decades, largely due to the low renin activity. This paper first describes the expression of the vasoconstrictive arm of RAS components in the brain as well as their physiological and pathophysiological significance. It then focus on the (pro)renin receptor (PRR), a newly discovered component of the RAS which has a high level in the brain. We review the role of prorenin and PRR in peripheral organs and emphasize the involvement of brain PRR in the pathogenesis of hypertension. Some future perspectives in PRR research are heighted with respect to novel therapeutic target for the treatment of hypertension and other cardiovascular diseases.

## 1. Introduction

 The renin-angiotensin system (RAS) plays an important role in the physiological and pathophysiological regulation of blood pressure (BP) and cardiovascular function. Renin, the rate-limiting enzyme of the RAS, was discovered more than a hundred years ago by Tigerstedt [1]. Angiotensinogen (AGT) is cleaved to angiotensin (Ang) I by renin, which is released from the juxtaglomerular apparatus. Ang I is then further converted into the octapeptide, Ang II, by the angiotensin-converting enzyme (ACE). Ang II is the main effector peptide in this system. Via binding to Ang II type 1 receptor (AT1R), Ang II stimulates vasoconstriction and secretion of the steroid hormone, aldosterone, which mediates sodium reabsorption and water retention [2]. In addition to the classical view of endocrine RAS, local RAS has been identified in various tissues, which interacts with the endocrine RAS [3–6]. 

 The first evidence for the existence of brain RAS was demonstrated by Brickerton and Buckley [7] who showed that administration of Ang II into the brain caused an increase in BP. It is well accepted now that RAS is intrinsic to the brain and plays an integral role in the pathophysiological regulation of cardiovascular function [8, 9]. The activities of brain RAS are achieved by influencing the autonomic nervous system, the baroreflex sensitivity, vasopressin (AVP) release, and the thirst and salt appetite [10–13]. Brain Ang II also acts as a neuropeptide via AT1R to increase the excitability of neurons in the cardiovascular regulatory centers of the hypothalamus and brainstem [14, 15]. Chronic central infusion of Ang II elicits an increase in arterial pressure and enhances the sensitivity of cardiac sympathetic afferent reflex via AT1R [16]. Human renin and AGT double transgenic mice exhibit Ang II-dependent hypertension, which can be attenuated by AVP antagonist suggesting an interaction between the brain RAS and AVP in hypertension [17, 18]. 

 Despite a multitude of evidence supporting the importance of Ang II action in the brain, conjecture remains about how Ang II forms locally in the brain because brain renin activity is undetectable using currently available methods [19, 20]. Prorenin has been recognized as a precursor of renin, but with very low angiotensinogenase activity [21]. Interestingly, we recently reported the existence of prorenin protein in brain tissues and the brain prorenin levels were ten-fold higher compared with renin levels [22]. The (pro)renin receptor (PRR) is a newly discovered component of the RAS which is able to promote Ang II formation via binding to renin or prorenin [23]. This timely discovery may shed new light on a possible pathway for Ang II formation in the brain regions behind the blood-brain barrier. Our paper focuses on the influence of brain RAS on the regulation of cardiovascular function with a specific emphasis on recent evidence concerning the role of brain PRR in the regulation of BP and cardiovascular homeostasis.

## 2. The Brain RAS

 Most brain areas are separated from the circulation by the blood-brain barrier which is impermeable to Ang II [24]. Therefore, Ang II needs to be generated locally or transported via transcytosis mechanism [25] to interact with its receptors located on neurons and astrocytes of the central nervous system (CNS). Genetic and pharmacological studies have demonstrated the intrinsic existence of brain RAS components [8, 9]. Furthermore, the pathophysiological significance of a brain RAS is supported by observations of increased RAS activities in the brain cardiovascular regulatory areas of hypertensive animal models [26, 27].

## 3. Renin 

 Renin is the rate-limiting enzyme for the formation of Ang I. Renin-like enzymatic activity within the brain was first reported by Ganten et al. [28]. However, the isolation of detectable active renin from the brain has remained unsuccessful for decades after its discovery [29]. Although the brain contains a large amount of enzymes, such as cathepsins [30] and tonin [31], which can also generate Ang peptides from AGT, the relevance of these alternative enzymatic pathways for Ang II generation in the brain remains elusive [32]. The most direct evidence supporting renin gene expression in the brain has come from Dr. Sigmund's group [33] who utilized an enhanced green fluorescence protein (eGFP) gene as a reporter for renin expression. They found renin promoter activity in the neurons of the cerebellum and hippocampus and in the cardiovascular regulatory regions such as the rostral ventrolateral medulla (RVLM), the subfornical organ (SFO), the paraventricular nucleus (PVN), and the supraoptic nucleus (SON). This group also discovered two different forms of renin in the brain, the intracellular (icRenin) and secret (sRenin) renin, derived from two different renin transcripts [34]. The relevance of icRenin is still unclear. This renin is a truncated form of prorenin lacking the first third of the prosegment, which remains intracellularly, whereas the sRenin secrets to the extracellular space. Due to the lack of machinery to cleave prorenin to renin in the brain [35], the sRenin in the brain is possibly prorenin. Although true renin activity exists at a finite level in the brain, trypsin or acid treated brain extracts showed a marked increase in renin activity. In addition, the inactive form of renin has been successfully isolated from the brain which could be activated by trypsin and inhibited by antirenin antibody *in vitro *[36]. This inactive form of renin may be the main source of renin-like activity in the brain. Pharmacological studies have provided strong support for the renin signal in the brain. For example, despite the difficulty of directly measuring renin activity in brain tissue, intracerebroventricular (ICV) infusion of the renin inhibitor, aliskiren, prevented sympathetic hyperactivity and hypertension as well as desensitization of arterial baroreflex function in Dahl salt-sensitive rats on a high salt diet [37]. These *in vivo* studies provide support for the existence of renin or renin-like activity in the brain and its role in hypertension. 

## 4. Angiotensinogen and ACE

 In the CNS, the main source of AGT synthesis is from the astroglia [38]. However, AGT expression has also been found in pure neuron cultures [39]. The AGT protein and its transcripts have been reported in the cardiovascular regulatory regions of the brain by different groups [40–42]. Transgenic rats expressing an antisense RNA against AGT mRNA specifically in the brain exhibited lower BP, polyuria, and reduction in plasma AVP [43] indicating that AGT is synthesized in brain and plays an essential role in BP regulation. 

 The ACE is a zinc metalloprotease which hydrolyzes the carboxyl terminal dipeptide His-Leu of Ang I to form Ang II. High densities of ACE were visualized in the choroid plexus, SFO, the caudate putamen, and the substantial nigra by autoradiography [44]. The ACE activity is present in the renin-containing synaptosomes of the neurons suggesting that intraneuronal synthesis of Ang I and Ang II is possible in synaptosomes [45]. The ICV delivery of the human ACE gene increased sympathetic activity, BP, and heart rate which were accompanied by increased Ang II and AVP production. These effects were abolished by ICV administration of an ACE inhibitor suggesting the importance of brain ACE in Ang II formation [26].

## 5. Angiotensin II and Angiotensin II Type I Receptor 

 The actions of Ang II are mediated predominantly by a seven transmembrane domain Gq-protein coupled receptor designated as AT1R. Effects of the AT1R are mediated by multiple intracellular signaling pathways, starting with G-protein and phospholipase activation, followed by an increase in intracellular inositol trisphosphate and calcium, which result in vasoconstriction, cell proliferation, and fibrosis [46, 47]. The AT1R is localized with high densities in the anterior pituitary, area postrema, lateral geniculate body, inferior olivary nucleus, median eminence, nucleus of the solitary tract (NTS), the anterior ventral third ventricle region, PVN, SON, and SFO [48]. The AT1R expression levels are tightly regulated by cardiovascular status and Ang II levels. Higher levels of AT1R were found in the hypothalamus of spontaneously hypertensive rat (SHR) and the SFO, PVN, and NTS of animals with chronic heart failure [27, 49]. Others have shown that chronic Ang II infusion upregulates AT1R mRNA and protein levels in the PVN and SFO via activation of intracellular mitogen-activated protein kinase (MAPK) signaling pathways [50]. Constitutive AT1R overexpression in the astroglia of RVLM resulted in a chronic increase in BP indicating that increased AT1 receptor activity is a primary determinant of efferent drive from RVLM [51]. Although Ang II is a small octapeptide, it is unable to permeate the blood-brain barrier and the blood-cerebrospinal fluid barrier [24, 52]. However, Ang II immunoreactivity has been found in the cell bodies of magnocellular and parvocellular neurons in the PVN and the magnocellular neurons in the SON [53] and Ang II-stained fibers have been found at all levels of the CNS, from the olfactory bulbs to the spinal cord. Furthermore, brain Ang II content was significantly increased in bilaterally nephrectomized rats despite diminishment of plasma Ang II to a very low level [54]. The body of evidence suggests that brain Ang II is synthesized locally and can be regulated independently of peripheral Ang II.

## 6. Prorenin 

 Prorenin, the precursor of renin, is cleaved to its active form by the removal of the 43 amino acid prosegment [21]. Whereas the renal juxtaglomerular cells constitute the most important source of circulating renin [55], a number of extrarenal tissues including the adrenal glands, ovary, testis, placenta, and retina produce prorenin [56]. This also explains the finding that the plasma prorenin level is 10 fold higher than that of renin [57]. However, prorenin has very low enzymatic activity in the plasma. Only two percent of prorenin exists as the “open form”, in which the prosegment of prorenin undergoes a conformational change and exposes the enzyme's active site [58]. This process is also called nonproteolytic activation of prorenin. The role of non-proteolytic activation of prorenin remains debatable due to the controversial phenotype in prorenin transgenic animal models. Liver-specific transgenic rats with a 400-fold increase in circulating prorenin exhibited severe renal lesions and hypertrophic cardiomyocyte with normal BP [59]. In contrast, transgenic animals with inducible or constitutive overexpression of prorenin, despite expressing 13–179 folds higher circulating prorenin, did not display cardiac or kidney damage. However, they did develop moderate Ang II dependent hypertension, since the BP was reduced by an ACE inhibitor [60, 61]. The reason for the presence of different phenotypes in the prorenin transgenic animal model remains unclear but could be due to the difference in the levels of plasma prorenin or the species. Although Mercure et al. [61] showed that Ang II is responsible for the increase in BP in the prorenin transgenic mice; the high plasma prorenin levels present in his study might not exist in physiological or even pathophysiological states [62]. The formation of Ang II in Mercure's model may be due to the activity of the two percent “open form” prorenin in the plasma [63]. However, in all three models, the increase in prorenin levels is limited to the circulation and liver. Thus, the role of local or tissue prorenin in cardiovascular regulation remains inconclusive. 

## 7. (Pro)renin Receptor

 In the year 2002, a new component of RAS was identified in human mesangial cells and named the (pro)renin receptor (PRR) because PRR binds both renin and prorenin [23]. The PRR gene is identical to ATPase 6 accessory protein2 (ATP6AP2) and is located on the X chromosome. PRR gene encodes a 350-amino-acidand ubiquitously expresses a single transmembrane protein with a large N-terminal extracellular domain which binds both renin and prorenin with affinities in the nanomolar range [64, 65]. Immunofluorescence observed by confocal microscope demonstrated that PRR was located on the cell surface, as well as intracellular compartments especially on the perinuclear space [22, 23, 66]. *In vitro* binding studies have shown that prorenin binds to PRR with a 3-4 fold higher affinity than renin, indicating that prorenin is a preferred ligand for PRR compared with renin [64, 65]. The binding of renin to PRR increases enzymatic activity 5-fold higher than the nonreceptor-bound renin [23]. The binding of prorenin to PRR induces a conformational change; the prosegment is removed from the catalytic cleft and the active site is accessible to AGT leading to full nonproteolytic activation of prorenin [23]. Interestingly, this phenomenon is reversible and prorenin eluted from the receptor reverts to its inactive form. The binding of renin to PRR is independent of the active site and receptor-bound renin or prorenin is not internalized or degraded [64, 67]. Thus, the discovery of the PRR has shed light on an alternate pathway for nonproteolytic activation of prorenin. Although the formation and role of intracellular Ang II has been previously reported [68, 69], there is a lack of attention to whether the intracellular PRR and prorenin contributes to the intracellular Ang II formation. It is likely that in tissues lacking a mechanism to cleave prorenin to renin, activation of prorenin via binding to PRR may contribute to intracellular Ang II formation. Several *in vitro* studies have been completed to assess the ability of prorenin to form Ang II via binding to the PRR. At a concentration of nanomolar range, which is much higher than the circulation level in physiological conditions, prorenin binds to PRR and exhibits enzymatic activity similarly to that of renin [64]. Furthermore, a recent *in vitro* study showed that the PRR activation by prorenin to generate Ang II requires about 800 fold higher prorenin concentration above normal plasma levels; the Ang II-independent activation requires an even higher prorenin concentration [70]. Combining these observations, prorenin may act mainly in the tissues either extracellularly or intracellularly, where its concentration may be high enough to activate Ang II-dependent or independent signals [70]. 

Transgenic rats with human PRR expression in vascular smooth muscle cells have been shown to exhibit hypertension and increased plasma aldosterone at six months, suggesting a pathological role of PRR in raising BP [71]. This study speculated that the rise in BP may have been caused by increased plasma aldosterone levels in these rats, but whether the increase in aldosterone depends on Ang II was not directly tested by the authors. In another transgenic model, ubiquitous expression of human PRR in rats resulted in proteinuria, glomerulosclerosis, MAPK activation, and cyclooxygenase-2 upregulation. These rats exhibited normal BP and renal RAS activity suggesting an Ang II independent nephropathy [72]. A previous *in vitro* study has shown that rat prorenin/renin was able to activate human PRR [65]. The phenotype of these transgenic rats could be due to the activation of human PRR transgenes by endogenous rat prorenin/renin. The reason for the differences in the phenotypes of these two models is not clear, but could be attributed to tissue targeting strategy differences. However, these studies clearly suggest that the pathological changes seen in these models might be due to the non-RAS dependent PRR rather than RAS dependent effects. In addition, binding of renin or prorenin to the PRR directly triggered intracellular MAPK signaling cascades in several cell types, which up-regulated the expression of profibrotic genes such as plasminogen activator inhibitor type (PAI)-1, collagens, and fibronectin [73–75]. Despite Ang II type 1 and 2 receptor blockade, renin or prorenin was still able to induce a long-lasting ERK 1/2 phosphorylation by binding to PRR, which was not blocked by aliskiren. Since aliskiren is a renin inhibitor which blocks the breakdown of angiotensinogen by renin, but does not affect the binding of renin to PRR and the PRR mediated signaling, it is clear that PRR mediates RAS independent signaling pathways [73]. 

 Global PRR knockout is lethal in mice indicating an essential role of PRR in embryonic development [76]. Recently, several tissue specific PRR knockout mouse models have been generated; [77–79] these *in vivo* studies provide additional insight into functions of PRR which are independent of RAS activation. PRR knockout in cardiomyocytes of mice led to heart failure and death within four weeks after birth accompanied by deacidification of the intracellular vesicles [77]. This outcome was also reported in the mice with a PRR deletion in the podocytes [78, 79]. In both cases, the defect appears to be associated with an inability to acidify intracellular compartments and a dysfunction of vATPase, suggesting a PRR effect unrelated to RAS *in vivo*. Moreover, PRR functions as a physiological adaptor between vATPase and the Wnt receptors [80]. Wnt via binding to its receptor, low-density lipoprotein receptor-related protein 6 (LRP6), induces receptor aggregation and phosphorylation of LRP6, resulting in the stabilization of *β*-catenin. The Wnt/*β*-catenin signaling is fundamental for a normal patterned embryo. In the adult, however, Wnt signaling is involved in cell proliferation and tissue homeostasis and has been implicated in certain pathologies, such as cancer and diabetes. By employing a genome-wide small inhibitory RNA (siRNA) screen, Cruciat et al. [80] identified PRR as part of the Wnt receptor complex, acting as a specific adaptor between LRP6 and vATPase where PRR and vATPase mediated Wnt signaling during anteroposterior patterning of Xenopus early CNS development. 

## 8. The PRR in the CNS

**Figure 1:**
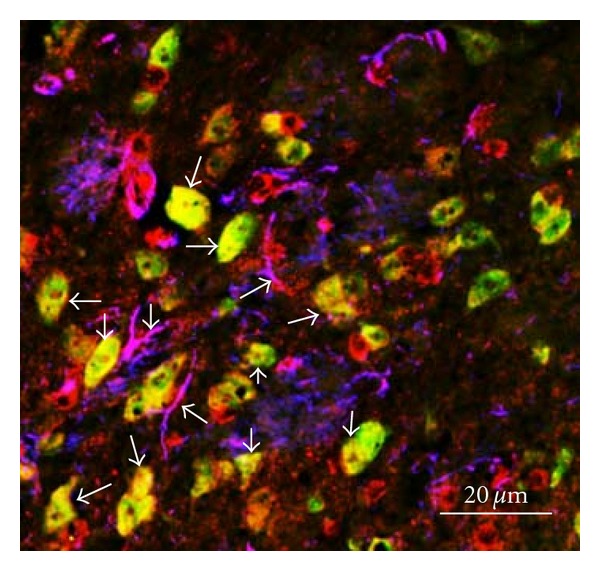
(Pro)renin receptor expression in the C57Bl/6J mouse brain. Triple immunofluorescence staining for PRR (red), neuron-specific nuclear protein (green, neuron marker), and glial fibrillary acidic protein (blue, astroglia marker) revealed that PRR is expressed in both neurons and astroglia with prominent expression in the neurons. Arrows indicate the colocalization of PRR with neurons or astroglia.

 A mutation of the PRR gene, resulting in frame deletion of exon 4 is associated with X-linked mental retardation and epilepsy pointing to an important role of PRR in the CNS [81]. PRR mRNA is widely expressed in various regions of the human brain with the highest expression levels in the pituitary and frontal lobe [82]. Immunostaining showed that PRR is colocalized with oxytocin and AVP in the magnocellular neurons of the PVN and SON indicating that PRR may be related to the central control of water-electrolyte homeostasis and BP [82]. Similarly, a wide distribution of PRR mRNA was found in key regions of the mouse brain involved in the regulation of BP and body fluid homeostasis [83]. Furthermore, PRR protein is expressed throughout the brain in cardiovascular regions including the SFO, PVN, nucleus of raphe pallidus, NTS, and RVLM as well as in noncardiovascular regulatory regions [22]. Immunofluorescence staining for PRR, neuron-specific nuclear protein (NeuN, neuron marker), and glial fibrillary acidic protein (GFAP, astroglia marker) revealed that PRR is expressed in astroglia, but with prominent expression in the neurons (Figure 1). Neuronal cells from the hypothalamus and brainstem of normotensive rat brains express PRR with levels 3-fold higher than that seen in astroglial cells from the same brain areas [66]. Moreover, PRR mRNA and protein levels were increased in brain cardiovascular regulatory regions in hypertensive animals [22, 84]. Knockdown of PRR in the SON was associated with attenuation of hypertension and a decrease in plasma AVP in SHR [84]. We recently reported that PRR knockdown in the brain attenuated Ang II-dependent hypertension in human renin and AGT double transgenic mice [22]. This effect was associated with a decrease in AVP levels, sympathetic tone, and improvement of baroreflex sensitivity indicating a role of PRR in the autonomic regulation of hypertension. However, whether PRR regulates BP and cardiovascular homeostasis via Ang II dependent or independent pathways remains inconclusive. 

**Figure 2:**
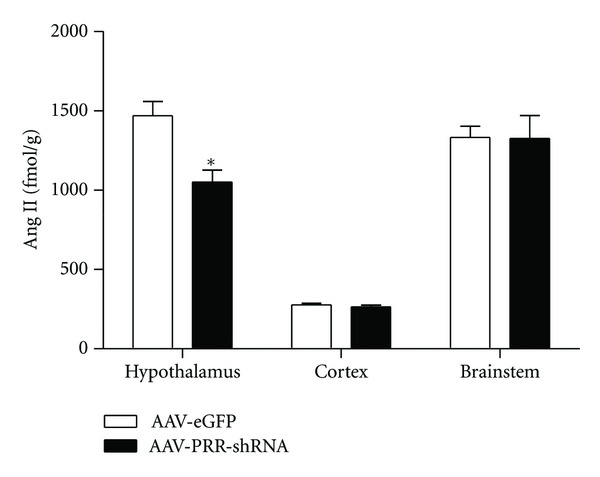
ICV delivery of (pro)renin receptor shRNA reduces brain Ang II level in the hypothalamus. The human renin and AGT double transgenic mice were ICV injected with AAV-PRR short hairpin RNA (AAV-PRR-shRNA, 3.5 × 10^11^ Vg/100nl) or control virus. Two weeks after virus injection, brain tissues were harvested for analysis of Ang II levels in the hypothalamus, cortex, and brainstem. (*n* = 6/group). *P < 0.05 versus AAV-enhanced green fluorescent protein (AAV-eGFP) treatment.

 Shan et al. [84] demonstrated that coincubation of human prorenin and AGT in isolated neurons evoked a dose- and time-dependent increase in Ang I and II formation indicating the ability of prorenin to generate angiotensin peptides in neurons possibly by binding to PRR. To address whether PRR mediates Ang II formation in the CNS, our laboratory recently measured Ang II levels in human renin and AGT double transgenic mice following brain-targeted PRR knockdown using PRR short hairpin RNA; Ang II levels were significantly decreased after PRR knockdown in the hypothalamus (Figure 2) and the levels were associated with a decrease of PRR levels in this region, as reported previously [22]. These data show that in hypertension, PRR may be involved in Ang II formation in the CNS. We also recently reported that the prorenin protein exists in mouse brain tissue, and its expression level is 10-fold higher than that of renin. In light of the facts that (1) there is abundant PRR expression in the brain, (2) prorenin exists in the brain despite an extremely low renin activity, and (3) manipulation of PRR modulates the Ang II levels; we propose that the binding of prorenin to PRR may initiate the rate-limiting step for angiotensin peptides formation in the CNS. The limitation of the knock down-based hypothesis is that PRR also acts as an accessory protein for vATPase, which is important for vesicular acidification, an important function in neuronal cells. Although the role of vATPase in Ang II formation has not been reported, it is difficult to distinguish the effects of PRR silencing on RAS-related prorenin inactivation and vATPase dysfunction. So far, the role of RAS independent signaling pathways of PRR in the CNS remains unknown. 

## 9. Conclusion and Perspective

 Emerging evidence supports the argument that brain RAS signals contribute to the development of hypertension. Interruption of these signals is beneficial to the control of BP in hypertension. All components of the RAS exist in the brain, but the renin level is extremely low. Prorenin and PRR may be the missing piece of the puzzle of the brain RAS since prorenin is conferred a nonproteolytic activation when binding to the PRR. Although further study is needed to test this hypothesis, evidence which support this case include that: (1) prorenin is the dominant form of total renin (prorenin and renin) in the brain, (2) PRR is highly expressed in the CNS, (3) brain PRR expression levels are increased in several hypertensive animal models, (4) knockdown of the PRR in the hypothalamus is associated with reduction in Ang II formation in this region during hypertension, and (5) reduced PRR expression level is associated with reduced BP in Ang II-dependent hypertension. Conversely, the RAS-unrelated PRR function via vATPase is critical to acidification of the intracellular compartments and might be important in the modulation of autonomic function since vATPase is responsible for neurotransmitter transportation and storage in the neuron vesicles [85]. We conclude that PRR might be an essential component of the tissue RAS, at least in the CNS. On the other hand, the RAS-unrelated PRR function in the CNS needs further investigation. Thus, targeting PRR can be an innovative, new strategy for the treatment of hypertension. Understanding the physiological and pathophysiological role of PRR in hypertension models would move this possibility forward. Because the PRR plays an essential role in embryonic development, generation of PRR antagonist or inducible tissue-specific PRR knockout animal models would be ideal tools to study the role of PRR in adult diseases models.
